# Effects of proactive population-based nephrologist oversight on progression of chronic kidney disease: a retrospective control analysis

**DOI:** 10.1186/1472-6963-12-252

**Published:** 2012-08-15

**Authors:** Brian Lee, Marianne Turley, Di Meng, Yvonne Zhou, Terhilda Garrido, Alan Lau, Linda Radler

**Affiliations:** 1 Division of Nephrology, Kaiser Permanente Hawaii, Moanalua Medical Center, 3288 Moanalua Rd, Honolulu, HI, 96819, USA; 2 HIT Transformation/Analytics, Kaiser Permanente, 500 NE Multnomah St, Portland, OR, 97232, USA; 3 HIT Transformation/Analytics, Kaiser Permanente, 1800 Harrison, Oakland, CA, 94612, USA

## Abstract

**Background:**

Benefits of early nephrology care are well-established, but as many as 40% of U.S. patients with end-stage renal disease (ESRD) do not see a nephrologist before its onset. Our objective was to evaluate the effect of proactive, population-based nephrologist oversight (PPNO) on chronic kidney disease (CKD) progression.

**Methods:**

Retrospective control analysis of Kaiser Permanente Hawaii members with CKD using propensity score matching methods. We matched 2,938 control and case pairs of individuals with stage 3a CKD for the pre-PPNO period (2001–2004) and post-PPNO period (2005–2008) that were similar in other characteristics: age, gender, and the presence of diabetes and hypertension. After three years, we classified the stage outcomes for all individuals. We assessed the PPNO effect across all stages of progression with a *χ*^2^- test. We used the z-score test to assess the proportional differences in progression within a stage.

**Results:**

The progression within the post-PPNO period was less severe and significantly different from the pre-PPNO period (p = 0.027). Within the stages, there were 2.6% more individuals remaining in 3a in the post-period (95% confidence interval [CI], 1.5% to 3.8%; *P* value < 0.00001). Progression from 3a to 3b was 2.2% less in the post-period (95% [CI], 0.7% to 3.6%; *P* value = 0.0017), 3a to 4/5 was 0.2% less (95% CI, 0.0% to 0.87%; *P* value = 0.26), and 3a to ESRD was 0.24% less (95% CI, 0.0% to 0.66%, *P* value = 0.10).

**Conclusions:**

Proactive, population-based nephrologist oversight was associated with a statistically significant decrease in progression. With enabling health information technology, risk stratification and targeted intervention by collaborative primary and specialty care achieves population-level care improvements. This model may be applicable to other chronic conditions.

## Background

In tandem with increasing rates of obesity and diabetes, the prevalence of chronic kidney disease (CKD) continues to rise. Recent estimates suggest that 11.5% to 14.5% of the U.S. adult population has early- to late-stage CKD [1-3]. Benefits of early nephrology care for CKD are well-established: slowed progression of disease, better preparation for renal replacement therapy (RRT), greater likelihood of outpatient hemodialysis initiation, and reduced mortality [4-6]. Yet as many as 40% of patients in the United States do not see a nephrologist before the onset of end-stage renal disease (ESRD) , and fewer than 25% have been followed by a nephrologist for at least a year before end-stage disease [7].

A new model of care providing proactive population-based nephrologist oversight (PPNO) reduces late referrals and increases the proportion of patients starting hemodialysis with a mature arteriovenous fistula and as outpatients [8]. The model consists of risk stratifying a CKD population and providing unsolicited nephrologist consultations to primary care physicians regarding individual patients, enabled by an integrated electronic health record (EHR). Nephrologists collaborate with primary care colleagues, helping primary care providers manage the patients they can and refer those patients who need specialist care. Our objective in this quality improvement project was to assess the effect of PPNO on CKD progression.

## Methods

### Setting

Kaiser Permanente Hawaii (KPHI) is one of eight regions of Kaiser Permanente, one of the United States’ leading health care providers and not-for-profit health plans with 9.0 million members in nine states and the District of Columbia. Kaiser Permanente addresses all health care needs for adults and children, including preventive, routine, specialty, emergency, and inpatient care, ancillary testing, pharmacy and rehabilitative services, and home care and functions as an accountable care organization [9]. KPHI has 224,000 members; 58 family practice physicians and 52 internists refer CKD patients to a specialty division with six nephrologists. KPHI has an integrated electronic health record (EHR), KP HealthConnect^TM^, providing comprehensive clinical information on all members.

### Population

Using the EHR, we identified all KPHI members during 2001–2010 who had at least two recorded serum creatinine levels a minimum of three months apart identifying different CKD stages and were at stage 3a CKD or higher. From this dataset, we excluded patients with serum creatinine patterns suggesting acute kidney injury: a decline in function within 14 days of a previous creatinine level followed by reversion to a previous higher level within 90 days. Patients whose renal function declined within 14 days of a previous creatinine level but who did not recover function within 90 days were included. In addition, we excluded those who had CKD or needed renal replacement therapy before 2001 and those whose CKD was diagnosed outside the KPHI system. Over the entire time period, we identifed 25,881 members with at least one stage of CKD.

### Proactive, population-based nephrologist oversight

In collaboration with primary care, nephrologists (B.L. and A.L.) developed a model of proactive population-based nephrology oversight (PPNO) for CKD patients. The model addressed both patients without referrals and those referred to nephrology by primary care physicians (PCPs).

#### Patients without referrals

1. Using data extracted from the EHR, the population management nephrologist (B. L.) generated concise monthly individual profiles for all KPHI patients with CKD, presented in risk-stratified order. Stratification criteria included glomerular filtration rate (GFR) and urine protein-to-creatinine ratio. The nephrologists identified specific laboratory criteria for patients at high risk of progression to ESRD: (1) GFR < 20 ml/min/1.73 m^2^, (2) GFR < 40 ml/min/1.73 m^2^ and urine protein-to-creatinine ratio > 2; or (3) urine protein-to-creatinine ratio > 4. Laboratory criteria for low risk status were GFR ≥ 30 ml/min/1.73 m^2^ and <1 gram proteinuria.

Weighting proteinuria equivalently with GFR in determining risk status runs counter to stage based management of CKD [10], but it was derived from internal data analysis and is in agreement with research [11-14]. An internal evidence-based guidelines committee reviewed and adopted the risk criteria and developed and distributed updated CKD treatment guidelines to all PCPs to support the quality improvement initiative [15].

2. Recognizing the impact of other factors, such as age and comorbid conditions, on the potential value of specialty care, the population management nephrologist reviewed comprehensive clinical information in the EHR for each high risk patient. Using clinical judgment to determine which patients would most benefit from nephrologist care, he contacted the responsible PCPs and requested those patients be referred for a nephrology visit.

3. For patients at low risk of progression, the nephrologist proactively provided electronic consultations to PCPs to improve CKD management, again using comprehensive information in the EHR. For example, the nephrologist advised primary care providers about maximal angiotensin-converting enzyme inhibitor or angiotensin II receptor blocker pharmacotherapy for patients with overt proteinuria and uncontrolled blood pressure. Management advice was sent to PCPs via KP HealthConnect messaging. Pre-programmed blocks of text minimized the time required to generate messages.

#### Referred patients

1. The population management nephrologist comprehensively reviewed the EHR for all patients referred for specialty care.

2. If the nephrologist judged the patient to be at low risk, he contacted the referring physician about the possibility of managing the patient in the primary care setting with specific management advice and the assurance that the patient would be electronically monitored by the nephrologist.

3. To ensure patient safety and quality of care, the population management nephrologist flagged these patient records and looked at them first when reviewing the monthly risk-stratified profiles.

Between 2004 and 2010, the population management nephrologist reviewed the profiles of the risk-stratified CKD population, approximately 15 patients per week, reviewing the KP HealthConnect record for additional information as needed. Among the reviewed cases, many patients benefitted from improved medical management, but most did not immediately need a referral. Model-identified consultations generated about 47 referrals per year of high-risk patients. Nephrologists and primary care providers collaboratively agreed to return about 25 low-risk referrals per year to primary care with active nephrologist surveillance.

### Design

We conducted a retrospective control analysis to quantify the rate of CKD progression between stages before and after the initiation of PPNO in 2005. We defined the pre-PPNO period as January 1, 2001 to December 31, 2004 and the post-PPNO period as January 1, 2005 to December 31, 2008.

### CKD staging, covariates, and data sources

We calculated an estimated GFR for each creatinine level for all patients using the Modification of Diet in Renal Disease Study equation, which incorporates creatinine level, age, gender, and race or ethnicity [16]. We did not have race or ethnicity data, so our calculations underestimate the true GFR scores for African Americans by a factor of 1.2. However, KP Hawaii has a very small African American population (1.0%), and we assumed this bias to be negligible. For patients with estimated GFRs < 60 ml per minute per 1.73 m^2^, we identified the applicable stage of CKD using a modified National Kidney Foundation classification of chronic kidney disease: 45 to 59 ml per minute per 1.73 m^2^ (stage 3a), 30 to 44 ml per minute per 1.73 m^2^ (stage 3b), 15 to 29 ml per minute per 1.73 m^2^ (stage 4), and less than 15 ml per minute per 1.73 m^2^ (stage 5) [17]. In addition, we identified patients receiving renal replacement therapy as being in ESRD.

In addition to data on CKD stage, we included the following covariates: patient demographics (age, gender, mortality), KP membership eligibility, diabetes, and hypertension. We collected serum creatinine level, patient demographics, and eligibility data from KP HealthConnect from 2001 to 2008. We collected data on diabetes and hypertension from Healthcare Employer Data and Information Set (HEDIS) data from 2001 to 2008.

### Statistical analysis

We used propensity score matching to control for potential sampling bias in our assessment of the effect of PPNO on the CKD population. For the entire population, we performed data cleaning and all statistical analyses using SAS 9.1 software. We pooled the data for stages 4 and 5 to increase sample size. We summarized the pre-matched population on the covariates for the entire population.

We identified patients diagnosed with stage 3a CKD in 2001 and 2002 as the controls and followed their disease progression for three years (2001 followed in 2001, 2002, and 2003; 2002 followed in 2002, 2003, and 2004). We identified patients diagnosed in 2005 and 2006 as the cases and followed their disease progression for three years (2005 followed in 2005, 2006, and 2007; 2006 followed in 2006, 2007, and 2008). Patients were identified by the year in which they had a first creatinine level and subsequent level within 90 days that quantified them as being in stage 3a, resulting in independent samples. To account for transient fluctuations in renal function, any change in estimated GFR sustained over 90 days was counted as progression.

We then matched control and case pairs, matching randomly one-to-one without replacement for the pre- and post- PPNO groups, based on propensity scoring for age, gender, and the presence of diabetes and hypertension. We summarized the matched data on the covariates and assessed the success of matching by examining absolute differences between the covariate distribution proportions in cases and controls.

Stage outcomes were observed for each matched individual remaining in stage 3a or progressing from stage 3a to stages 3b, 4/5, and ESRD at one, two, and three year intervals in the pre- and post-PPNO periods. We tallied the final stage outcomes at the end of three years. We tested the overall effect of the PPNO on stage outcomes using the *χ*^2^-test. Within stages, we converted the counts to proportions and assessed the differences between the pre- and post-PPNO periods with z-score tests.

The Kaiser Permanente Hawaii Institutional Review Board approved the project.

## Results

### Analysis population

We identified 4,087 members at stage 3a in 2001 and 2002; 46% were male and 63% were 65 years of age or older (Table 1). We identified 3,453 members at stage 3a in 2005 and 2006; 46% were male and 59% were 65 years of age or older.

**Table 1:** Characteristics of the analysis population and pre- and post-intervention groups for pre-matched and post-matched data

	**Pre-matching**		**Post-matching**	
	**Pre-intervention N = 4,087**	**Post-Intervention N = 3,453**	**Pre-intervention N = 2,958**	**Post-Intervention N = 2,958**
Female	2,228 (54.5%)	1,875 (54.3%)	1,603 (54.2%)	1,608 (54.4%)
Age				
0 - 19	1 (0.02%)	1 (0.03%)	1 (0.03%)	1 (0.03%)
20 - 44	223 (5.5%)	205 (5.9%)	182 (6.2%)	179 (6.1%)
45 - 64	1,276 (31.2%)	1,200 (34.8%)	965 (32.6%)	965 (32.6%)
≥ 65	2,587 (63.3%)	2,047 (59.3%)	1,810 (61.2%)	1,813 (61.3%)
Diabetes	844 (20.7%)	111 (3.2%)	111 (3.8%)	111 (3.8%)
Hypertension	1030 (25.2%)	683 (19.8%)	412 (13.9%)	415 (14.0%)

### Differences in CKD progression on matched data

We obtained 2,958 matches between the pre- and post-PPNO data; the matches were well-balanced on the covariates (Table 1). During the pre-PPNO period, members had an average of 8.52 serum creatinine levels recorded; during the post-PPNO period, the comparable figure was 9.07 (*P* = 0.049). The average first estimated GFR within stage 3a was 53.37 for members in the pre-PPNO period and 53.63 in the post-period; this difference was not statistically significant (*P* = 0.052).

Stage outcomes for all individuals after three years were tallied (Table 2). The progression within the post-PPNO period was less severe and significantly different from the pre-PPNO period (p = 0.027). Within stage 3a, 76 more individuals, or 2.6% of the match total, remained in the post-period (95% confidence interval [CI], 1.5% to 3.8%; *P* value < 0.00001). For stage 3b, 64 fewer individuals, or 2.2%, progressed from stage 3a to stage 3b (95% CI, 0.7% to 3.6%; *P* value = 0.0017). Six fewer individuals, or 0.20%, progressed from stage 3a to stage 4/5 (95% CI, 0.0% to 0.87%; *P* value = 0.26). Seven fewer individuals, or 0.24%, progressed from stage 3a to ESRD (95% CI, 0.0% to 0.66%, *P* value = 0.10). There was one more death, or 0.034%, in the post-period (95% CI, 0.0% to 1.19%; *P* value = 0.52).

**Table 2:** CKD stage outcomes after three years among pre- and post-PPNO groups of individuals initially identified as Stage 3a

**Stages**	**Pre-PPNO progression**		**Post-PPNO progression**		**Difference**	
	**Individuals (n = 2,958)**	**Proportion**	**Individuals (n = 2,958)**	**Proportion**	**Individuals**	**Proportion**
3a	2,438	82%	2,514	85%	−76	2.6%
3a to 3b	294	9.9%	230	7.8%	64	2.2%
3a to 4/5	49	1.7%	43	1.5%	6	0.20%
3a to ESRD	19	0.6%	12	0.4%	7	0.24%
Dead	158	5.3%	159	5.4%	−1	0.034%

## Discussion

The overall effect of proactive population-based nephrologist oversight was statistically significant. The post-PPNO period showed less severe progression. In particular, significantly more individuals remained in stage 3a after three years. Progression from stage 3a to 3b was significantly less in the post-period and marginally statistically significantly less from stage 3a to ESRD. Our finding that appropriate population care management for patients with CKD slows the progression of disease is consistent with other observational studies [18].

What our project adds to the literature is a model of specialists providing population-based care that identifies and risk stratifies the entire CKD population, directing nephrology resources to patients in need of them. With enough information provided by the electronic health record about individual cases, nephrologists can determine which patients need referrals and which can be managed by primary care physicians with nephrologist surveillance.

A similar model of systematic surveillance and shared management by specialty and primary care also resulted in slowed progression of disease [19]. However, our population was defined by having CKD, not by having received a referral for nephrology care. To the best of our knowledge, our report is the first to detail the benefits of population management by a specialty service of all CKD patients—those who have been referred for nephrologist care and those who have not.

Strengths of our report include the use of propensity score matching to control for identified confounders for which EHR data from 2001 to 2008 existed. These included diabetes, which we identified using HEDIS measures. HEDIS does not allow similar identification of members with cardiovascular disease (CVD). However, diabetes is a major risk factor for CVD [20], and we can reasonably assume that matching on diabetes status eliminates some of the potential confounding introduced by CVD status.

Members had more serum creatinine levels recorded during the post-PPNO period than in the pre-period. This is consistent with the goal of the program. In addition, it provides a perspective on our analysis as relatively conservative, since fewer levels during the pre-period could reflect some undetected progression from one stage to the next.

Several limitations deserve mention. Other factors may have contributed to the effects we observed. Two conferences in January 2005 and March 2007, sponsored by the KPHI nephrology department and attended by Kaiser Permanente and external physicians, focused on improving the management of patients with CKD. In addition, in June of 2007, an internal initiative was undertaken to improve hypertension management among patients with diabetes; chronic kidney disease is associated with diabetes, hypertension, or both in more than 90% of people with an estimated GFR below 60 ml per minute per 1.73 m^2^[21]. Finally, the use of panel management in primary care, facilitated by the Panel Support Tool, increased during the observation period [22,23]. The latter two factors were reflected in substantially improved HEDIS measures for calendar year 2007 (reported in 2008). Within the Medicare population, the percentage of patients with diabetes whose blood pressure was less than 140/90 mm Hg increased from 58.4% to 72.0%; the percentage whose blood pressure was less than 130/80 mm Hg increased from 36.3% to 44.3%.

We did not have comparable population data from the pre- and post-PPNO periods for corollary measures, such as angiotensin-converting enzyme inhibitor/angiotension II receptor blocker use or blood pressure control. Such data could provide more insight into the differences in progression.

Our use of an retrospective control group was an additional, albeit necessary, limitation to this evaluation. Randomizing members into PPNO or usual care was both inconsistent with the quality improvement nature of this initiative and would have reduced our ability to make statistical inferences, given the small population that progressed from stage 3a to a later stage of CKD. In addition, the Hawaii population is distinguished by a higher proportion of members of Asian/Pacific Islander origin, precluding the use of a control group from another region. We also limited the post-period to 2008 to have a balanced population with the pre-period.

**Figure 1:**
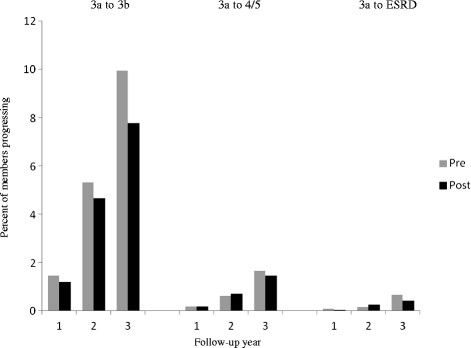
** Progression from stage 3a CKD at one to three years.** The percent of patients progressing from stage 3a to stage 3b, 4/5, and ESRD are shown for one, two, and three years of follow up.

Our population included only members with serial recorded serum creatinine levels; reduced GFR is undetected in a substantial minority of individuals with CKD [24]. Bias may have been introduced when PCPs selected patients for renal function testing. Our follow-up period of three years was likely too short to fully capture the effect of PPNO. The few existing reports of the natural history of CKD suggest that three years is a minimum follow-up period [25,26]. Figure 1 suggests an increasing effect with longer follow-up, although further assessment is needed to confirm this. Additionally, the lack of statistically significant decrease in progression from stage 3a to stage 4/5 may be due to sample size and to the decreasing slope of progression in the later stages of CKD [27]. In addition, data on the prevalence of diabetes in the pre- and post-PPNO populations were not optimal, but the impact was limited by the use of a more rigorous propensity score matching methodology.

Proactive population-based nephrologist care requires comprehensive health information technology. Our model of care is an example of its meaningful use to improve patient outcomes, in keeping with the spirit of the Health Information Technology for Economic and Clinical Health (HITECH) Act of 2009 [28]. In addition to generating a list of patients with CKD, which is an objective in the menu set of the final regulation, we used health information technology to risk stratify the population, communicate with primary care physicians, and review and record complete clinical information [29]. Our model of care provides additional evidence supporting the federal government’s investment in health information technology as a lever for improving health care quality and efficiency.

However, health information technology alone is insufficient to create change of the magnitude we observed. It is important to note that organization-wide goals and financial incentives are aligned throughout Kaiser Permanente, as an example of an accountable care organization, to provide efficient and effective care and to consider the larger health trajectory of the patient; neither nephrologists nor primary care physicians are penalized or rewarded for shifts in the volume of visits.

Risk stratification allows specialists to address all CKD patients regardless of referral status. This approach can potentially be applied to other chronic conditions in which at least one key metric related to quality of care is available. For instance, pain management specialists might use the number of dispensed opiate prescriptions to identify chronic pain patients with unmet needs, and cardiologists could use the ejection fraction to risk stratify the population of patients with congestive heart failure. Future research is needed to confirm this.

Future research is also needed to evaluate the cost-effectiveness of this approach. We estimated that approximately $8 million in RRT costs were avoided between 2005 and 2008 by the reduced progression to ESRD but did not take into account any other factors. The reduced progression from stage 3a to stage 3b may have no effect on RRT rates or mortality.

## Conclusions

Proactive, population-based nephrologist oversight was associated with a statistically significant decrease in progression. In accountable care settings with enabling comprehensive health information technology and aligned incentives, risk stratification and targeted proactive intervention can achieve population-level care improvements in chronic kidney disease. In the face of expanding demand for care under U.S. health care reform, our model provides evidence that health information technology can help leverage existing resources to provide better care for more people.

## Competing interests

All authors declare that they have no competing interests.

## Authors’ contributions

BL conceived of and implemented the model of care and revised the manuscript for important intellectual content. MT, DM, and YZ conducted data collection, analysis, and interpretation and were involved in drafting and revising the manuscript. TG and LR were instrumental in the conception and design of the evaluation and contributed important concepts to the manuscript. AL was involved in the conception and implementation of the model of care oversight and reviewed the manuscript for important intellectual content. All authors read and approved the final manuscript.

## Pre-publication history

The pre-publication history for this paper can be accessed here:

http://www.biomedcentral.com/1472-6963/12/252/prepub
